# Nutritional and antinutritional evaluation of complementary foods formulated from maize*,* pea, and anchote flours

**DOI:** 10.1002/fsn3.1516

**Published:** 2020-03-20

**Authors:** Habtamu Fekadu Gemede

**Affiliations:** ^1^ Food and Nutritional Science Department of Food Technology and Process Engineering Wollega University Nekemte Ethiopia

**Keywords:** anchote, antinutritional, complementary foods, maize, nutritional, pea

## Abstract

This study was aimed to evaluate nutritional and antinutritional contents of complementary foods from locally available and affordable raw materials (maize, pea, and anchote) grown in Western Ethiopia. The six formulated complementary diets analyzed for their proximate, mineral, and antinutritional continents were compared with Codex standards. The mineral ratios and molar ratios of the formulated diets were also evaluated and compared with each standard values. Six formulations were generated by d‐optimal mixture design. The formulated ingredient ranges 45%–61% maize, 23%–31% pea, and 14%–28% anchote. Design‐Expert^®^ 6 (Stat‐Ease) was used to constrain the three components. The formulated diets ranged from 14.92% to 20.99%, 5.95% to 9.94%, 2.75% to 3.41%, and 59.10% to 66.22% of protein, fat, fiber, and utilizable carbohydrate, respectively. Mineral contents (mg/100 g) of the formulated diet ranged from 225.45 to 261.32, 11.48 to 12.61, 2.73 to 3.00, 357.92 to 391.13, 298.55 to 332.63, 252.00 to 278.01, and 44.26 to 51.56 for calcium, iron, zinc, phosphorous, potassium, sodium and magnesium, respectively. The proximate and mineral contents of the formulated diet 5 meet the Codex standards, except the fat contents of the complementary food standards. The molar ratios of the formulated diets in this study were below standard reference and which show the high mineral bioavailability in all the formulated diets. The results of the study revealed that the formulated diets contain very low antinutritional factors and high mineral bioavailability. The paper's findings show that the complementary food formulated from maize*,* pea, and anchote flours particularly diet 5 may be suitable to alleviate protein energy malnutrition and it can be used as a substitute for the expensive commercial complementary food.

## Graphical abstract

Complementary food formulation from locally available food staffs.

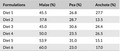

## 1 INTRODUCTION

Complementary food is a food either liquid or solid that can be given to infant and young children (6–24 months) along with breast milk (WHO, 2001). While the ages of children increase, the ability of breast milk to provide essential nutrient requirements decreases (Agostoni et al., 2008). In line with this, complementary food is the most determinant for the growth of the future children. Therefore, timely providing appropriate complementary foods during infancy is paramount for child growth in both nutritional and developmental reasons (Kamchan, Puwastien, Sirichakwal, & Kongkachuichai, 2004), but the quality of complementary foods to meet the required essential nutrient for children is very essential (Kamchan et al., 2004).

Cereal‐based complementary foods do not contain enough essential nutrients to meet daily requirements for infant and young children (WHO, 2001). The complementary feeds given to many developing country including Ethiopia are deficient in essential nutrients (Zewditu, Kelbessa, Timotewos, & Ayele, 2001). Therefore, to improving the nutritional status of children and mitigate protein energy malnutrition, the locally available essential nutrient‐rich complementary foods that are locally available and affordable should be encouraged (WHO/FAO, 2004).

On the other hand, in Ethiopia, the cost of animal product‐fortified complementary foods is not affordable for the rural community (Muhimbula, Issa‐Zacharia, & Kinabo, 2011). Hence, many poor‐resource families depend on inadequate complementary diets for their infant. This is the reason why undernutrition is a major public health problem in Ethiopia (WHO, 2001). Accordingly, insufficient complementary food is a main ground for the occurrence of infant malnutrition (Krebs & Westcott, 2002). To enhance these troubles, searching for alternative low‐cost complementary foods with high essential nutrients and that can be locally available for the community is the best strategies to improve infants' nutrition (Muhimbula et al., 2011; Temesgen, 2013). In line with this, many scholars tried to evaluate these problems, but still malnutrition vestiges a grave predicament (Zewditu et al., 2001) and economic encumber in the country (Muhimbula et al., 2011). Therefore, this study was aimed to formulate and evaluate the nutritional and antinutritional properties of locally available and affordable complementary foods from anchote (*Coccinia abyssinica*), pea (*Pisum sativum*), and maize (*Zea mays*) flours in Western Ethiopia.

## 2 MATERIALS AND METHODS

### 2.1 Description of sampling site

Maize, pea, and anchote samples were collected from Nekemte market. Nekemte, the capital town of East Wollega Zone is located 322 km away from capital city of the country, in the western part of the country. In East Wollega Zone, agriculture (crop cultivation and rearing of livestock) is the leading economic activity in the district. The food crops cultivated in the district are cereals (maize, sorghum, barley, teff, and finger millet), pulses (peas, beans, and nug), root and crops (anchote, Oromo potatoes, and sweet potatoes), fruits (lime, orange, mango, avocado, banana, papaya, and pumpkin), spices (cardamom, long pepper, chilies, and ginger), and vegetables (cabbage and mustard seed). In addition, rearing of livestock such as cattle, sheep, chicken, and donkeys is common.

### 2.2 Preliminary survey

To collect data from the respondent interview, semistructured questionnaire was prepared. Twenty‐two respondents were selected purposefully. Among them, twelve respondents were mothers who have complementary age children during the interview, six respondents were health extension and four respondents were agricultural expertise who possesses knowledge of complementary food production, marketing, and consumption of locally available complementary foods. The central theme of the interview was to gather prior information about production, handling, processing, and utilization of complementary food. The initial survey data lead the experimenter to have basic information on the type and production of locally available complementary food produced and to fix the commonly available complementary food for sample selection. Based on the respondent response, maize, pea, and anchote are the commonly used complementary food locally available in East Wollega Zone, Ethiopia. In addition, wheat and teff are also used for complementary food formulation. Based on this information, we have selected known variety of maize, pea, and anchote for the formulation of locally available foods in East Wollega Zone, Ethiopia.

### 2.3 Sample collection and preparation

Maize (BH66 variety, 2 kg), pea (karse variety, 2 kg), and anchote (dime, 6 kg) samples were purchased from the local market, Nekemte, Ethiopia. The collected samples were coded, packed, and prepared at Wollega University research laboratories of Food Technology department. The maize and pea samples were sorted and sun‐dried separately and then oven‐dried at 45°C. Anchote tuber was washed by distilled water, was sliced to uniform thickness of 5 mm using a stainless‐steel knife, and was sundried. All the dried samples were milled separately into a fine powder (0.425 mm sieve size). The moisture content of the samples was determined immediately after milled into fine powder. Finally, the powder was packed into airtight polyethylene plastic bag and was stored in a desiccator until required for further analysis. All chemicals used were of analytical grades.

### 2.4 Experimental design and treatment combinations

Table 1 shows the d‐optimal mixture design obtained from generated experimental design and the embarrassed area is shown in Table 2. Three mixtures were made from treatment including the following: 14 < anchote < 28, 45 < maize < 61, and 23 < pea < 31 (anchote + maize + pea = 100%). Therefore, from the treatment combinations, six samples were generated. A blend of these ingredients was therefore expected to give complementary foods of very balanced nutritional value.

**Table 1:** Ratios obtained by mixture design for the six formulations of flour

Formulations	Maize (%)	Pea (%)	Anchote (%)
Diet 1	45.5	26.8	27.7
Diet 2	57.8	28.7	13.5
Diet 3	45.0	30.6	24.4
Diet 4	50.0	23.5	26.5
Diet 5	53.9	31.0	15.1
Diet 6	60.0	23.0	17.0

**Table 2:** Restraint region of maize, pea, and anchote taken by mixture design

Low	≤Constraint	≤High
0.45	≤A: Maize	≤0.61
0.23	≤B: Pea	≤0.31
0.14	≤C: Anchote	≤0.28

### 2.5 Determination of proximate composition

#### 2.5.1 Determination of moisture content

The proximate composition analysis including moisture content, crude protein, crude fat, crude ash, and crude fiber was determined according to approved AOAC (2000) method. Utilizable carbohydrate content was calculated by difference, that is, 100 − (% crude protein + % crude fiber + % total ash + % crude fat). The gross energy content was determined by calculation from fat, carbohydrate, and protein contents using conversion factors; 4 kcal/g for protein, 9 kcal/g for fat, and 4 kcal/g for carbohydrates (Guyot, Rochette, & Treche, 2007).

#### 2.5.2 Determination of mineral content

The minerals were determined according to the standard method of AOAC (2000). Calcium, iron, and zinc were determined by using atomic absorption spectrophotometer (AAS), while sodium and potassium contents were determined using flame photometer (Jenway, PF 7). Phosphorus was determined by the colorimetric method using ammonium molybdate (AOAC, 1984).

#### 2.5.3 Determination of mineral ratios

The mineral ratios are often more important than individual mineral levels themselves because they are useful in determining nutritional interrelationships and also provide information regarding the many possible factors that may be represented by a disruption of their relationships such as disease states, physiological and developmental factors, and the effects of diets (Hoskin & Ireland, 2000). The mineral ratio was calculated by dividing the first mineral level to the second mineral level (Jacob, Etong, & Tijjani, 2015).

### 2.6 Determination of antinutritional factors

#### 2.6.1 Determination of phytate content

Phytate was determined by the method described by Vaintraub and Lapteva (1988). Oxalate was analyzed using the method originally used by Ukpabi and Ejidoh (1989). Tannin content was determined according to the method described by Maxson and Rooney (1972).

#### 2.6.2 Determination of molar ratio of antinutrients to minerals

The molar ratio was predicted by dividing the mole of antinutrient to the mole of minerals (Norhaizan & Norfaizadatul, 2009).

### 2.7 Statistical analysis

Mixture design using Design‐Expert^®^6 (Stat‐ Ease) was used to constrain three components. The completely randomized design was employed with two replicates. SPSS version 20.0 for windows was used to perform all the statistical analyses. One‐way analysis of variance (ANOVA) was used to evaluate the data. Duncan's multiple range test was used to separate means, and the result was reported as a mean ± standard error (*SE*). A *p*‐value of .05 or less was considered as the statistically significant difference.

## 3 RESULTS AND DISCUSSIONS

### 3.1 Proximate composition of the ingredients and formulated diets

Table 3 shows the percentage of proximate composition of the various ingredients (maize, pea, and anchote flours) used for the formulation of complementary foods in this finding. The moisture contents of the ingredients were 4.49%, 5.03%, and 6.91% for maize, pea, and anchote flours, respectively. Moisture content determination is key factors affecting the storage, shelf life, and safety of foods. The crude protein contents of the ingredients were 18.79%, 26.04%, and 12.20% for maize, pea, and anchote flours, respectively. Crude protein content of pea flour was significantly (*p* < .05) higher than maize and anchote flours. Akingbala, Akinwande, and Uzo‐Peters (2003) reported that pea contains appreciable protein, and this study also revealed that pea seeds are a good source of protein for potential for complementary food formulation. In addition, consumption of the seeds should be encouraged to mitigate protein energy malnutrition in the country.

**Table 3:** Proximate composition (%, dwb) and total energy (kcal/100 g) contents of maize, pea, and anchote flours

Ingredients	Moisture	Protein	Fat	Ash	Fiber	Util. CHO	Gross energy
Maize flour	4.49 ± 0.11^c^	18.79 ± 0.28^b^	12.86 ± 0.03^a^	2.54 ± 0.06^b^	2.51 ± 0.06^b^	58.81 ± 0.07^b^	426.14 ± 2.45^a^
Pea flour	5.03 ± 0.22^b^	26.04 ± 0.17^a^	2.59 ± 0.56^b^	2.56 ± 0.75^b^	1.92 ± 0.01^b^	61.86 ± 0.33^b^	374.91 ± 1.42^b^
Anchote flour	6.91 ± 0.03^a^	12.20 ± 0.04^c^	1.93 ± 0.39^c^	3.87 ± 0.45^a^	7.39 ± 0.04^a^	67.69 ± 0.45^a^	336.93 ± 2.41^c^

Note

Values are expressed as mean ± *SE*. Means not followed by the same superscript letters are significantly (*p* < .05) different.

The crude fat contents of the ingredients were 12.86%, 2.59%, and 1.93% for maize, pea, and anchote flours, respectively. Maize flour was significantly (*p* < .05) higher in crude fat content followed by pea and anchote flours. The ash stuffing of the component was 2.54%, 2.56%, and 3.87% for maize, pea, and anchote flours, respectively. Anchote tuber contained fairly high ash content which is an indication that anchote would provide essential minerals needed for body development. Anchote flour was also significantly (*p* < .05) higher in crude ash content than maize and pea flours. Crude fiber contents of the ingredients were 2.51%, 1.92%, and 7.39% for maize, pea, and anchote flours, respectively. Anchote flour was significantly (*p* < .05) higher in crude fiber content and followed by maize and pea flours. This finding revealed that anchote tuber is considered as a main source of crude fiber. Utilizable carbohydrate contents of the ingredients were 58.81%, 61.86%, and 67.69% for maize, pea, and anchote flours, respectively. The gross energy contents (kcal/100 g) of the ingredients were 426.14, 374.91, and 336.93 for maize, pea, and anchote flours, respectively. Gross energy contents of maize flour were higher than pea and anchote flours. This indicates that maize flour could be a major source of energy.

The proximate composition of the formulated diet is presented in Table 4. The range of moisture content of the six formulated diets was from 4.78% to 5.31%. Diet 3 having the blend of 30.6% pea, 24.4% anchote, and 45.0% maize were the highest in crude fiber while the lowest moisture contents were observed in diet 2 with the blend of 13.5% anchote, 28.7% pea, and 57.8% maize (Table 4). When the proportion of maize decreased and increased with anchote flour blend, the moisture content was increased. In this finding, the formulated diet 6, diet 5, and diet 2 convene the recommended moisture content (<5%) by CODEX CAC/GL (1991) (Table 4). Monitoring the moisture content in foods and food products is crucial because high moisture contents can reduce shelf life by increasing microbial degradation activity, resulting in bad odor and unacceptable taste of the product (Olu‐Owolabi, Fakayode, Adebowale, & Onianwa, 2007).

**Table 4:** Proximate composition (%, dwb) and gross energy (kcal/100 g) contents of the formulated complementary diets^a^

Formulated diets	Moisture content	Crude protein	Crude fat	Crude ash	Crude fiber	Utilizable carbohydrate	Gross energy
Diet 1	5.18	18.83	5.98	2.99	3.41	63.61	383.58
Diet 2	4.78	20.99	9.52	1.98	2.86	59.87	409.12
Diet 3	5.31	19.67	5.95	2.82	3.27	62.98	384.15
Diet 4	5.11	18.69	6.54	2.91	3.39	63.36	403.22
Diet 5	4.89	14.92	8.74	2.48	2.75	66.22	404.62
Diet 6	4.97	20.57	9.94	2.39	3.03	59.10	408.14
Codex standard^b^	<5	15	10–25	<3	<5	60–75	400–425

^a^ Values are expressed as mean ± *SE*. Means not followed by the same superscript letters are significantly (*p* ≤ .05) different.

^b^ CODEX CAC/GL 08 (1991).

The formulated diets contain 14.92%–20.99% crude protein contents. The highest crude protein was observed in diet 2 ratios, which contain 13.5% anchote, 28.7% pea, and 57.8% maize, while the least protein was observed in diet 5 that contain the blend of 15.1% anchote, 31.0% pea, and 53.9% maize. Protein content was decreased with increasing anchote proportion and increased with increasing pea and maize proportions (Table 4). The blending of cereal‐based foods can improve the protein content of the diets (Gibson & Hotz, 2001). The result is in agreement with Saeeda et al. (2009) who reported 17.5% crude protein from cabbage pea, groundnut, and wheat blends. The result is less than the value reported by Ijarotimi and Keshinro (2012) (23.85%–28.84%) who formulated complementary foods from fermented and germinated popcorn, but higher than the value reported by Bojňanská, Frančáková, Líšková, and Tokár (2012) (12.57%–16.28%) who formulated diets from lentil, chickpea, and wheat flour. The observed difference might be due to the difference in blending proportion and the type and varieties of crops used for the specific diet formulation. In this finding, all the formulated diets meet the recommended protein content (>15%) by CODEX CAC/GL (1991) (Table 2) and WHO/FAO (2004) (>15).

The crude fat content of the formulated diets ranged from 5.95% to 9.94%. The highest crude fat content was recorded in blend of 60.0% maize, 23.0% pea, and 17.0% anchote (diet 6), whereas the least was found in 45.0% maize, 30.6% pea, and 24.4% anchote blend (diet 3) (Table 2). Crude fat content was increased with increasing maize proportion and decreased with increasing pea flour proportion in the blend. Though, the result of this study was lower than the value reported by Solomon (2005) (11.5%–24.8%) who processed complementary diets from different cereal crop and vegetable products like maize, rice, soya beans, acha grains, benniseed, crayfish, carrot, bambara nut, and garden egg. WHO/FAO (2004) reported that the fat content of complementary diets should be ranged from 10% to 25% in which the result of the present study is less than the daily recommended fat content. This may be due to the less fat content of the raw materials used in the formulation of the food. In this finding, increasing the maize content and/or addition of oil can improve the fat content of the formulated complementary food.

The crude ash content of the six formulated complementary diets ranged from 1.98% to 2.99%. Diet 1 (27.7% anchote, 45.5% maize, and 26.8% pea) contained the highest total ash content while diet 2 (13.5% anchote, 28.7% pea and 57.8.0% maize) contained the least ash content. WHO/FAO (2004) recommended that the ash contents of complementary food should be less than five, in which all the formulated diets in the present study meet this recommended standards.

Crude fiber content of the six formulated diets ranged from 2.75% to 3.41%. The highest crude fiber content was recorded in blend of 45.5% maize, 26.8% pea, and 27.7% anchote (diet 1), whereas the least was found in 53.9% maize, 31.7% pea, and 15.1% anchote blend (diet 5). As shown in Table 4, the crude fiber content was decreased with increasing maize proportion and increased with increasing anchote in the blend. Ahmed, Bébbé, Clergé, Clément, and Mohammadou (2010) reported that anchote tuber is a potential source of fiber that can be used for physiological effects in human, as they stimulate and accelerate intestinal contraction and transit, and increase feces volume. The daily recommended allowance of crude fiber in the complementary food is <5% (CODEX CAC/GL, 1991). Consequently, all the formulated diets in the present finding meet the above‐stated standard.

Utilizable carbohydrate content of the six formulated complementary diets ranged from 59.10% to 66.22%. The highest utilizable carbohydrate was obtained in the ratio of 53.9% maize, 31.0% pea, and 15.1% anchote (diet 5), whereas the least was found in 60.0% maize, 23.0% pea, and 17.0% anchote blend (diet 6). All the formulated complementary foods except diet 2 and diet 6 in this work meet the carbohydrate content recommended by CODEX CAC/GL (1991) (60–75). Gross energy content of the formulated complementary diets in the present study was ranged from 383.58 to 409.12 kcal/100 g in the diet 1 and diet 2, respectively. All the formulated complementary foods except diet 1 and diet 3 in this work meet the gross energy content recommended by CODEX CAC/GL (1991) (400–425 kcal/100 g).

### 3.2 Mineral contents of the raw ingredients and formulated diets

Table 5 shows the mineral contents of the various ingredients (maize, pea, and anchote flours) used for the formulation of complementary foods. Calcium, sodium, and magnesium contents of anchote flour was significantly (*p* < .05) higher than that of maize and pea flours. However, the iron and zinc contents of the maize flour were significantly (*p* < .05) higher than pea and anchote flours. Minerals are essentially required for tissue functioning in human beings, and their presence in plants can have a positive contribution as a source of essential nutrients or even as active principles, or a negative effect because of the accumulation of high concentrations of potentially toxic elements (Olu‐Owolabi et al., 2007). Potassium and phosphorous is rich in pea flour. This indicates that all the samples are rich in different minerals.

**Table 5:** Mineral contents (mg/100g, dwb) of maize, pea, and anchote flours^a^

Ingredients	Calcium	Iron	Zinc	Phosphorous	Potassium	Sodium	Magnesium
Maize flour	246.16 ± 0.57^b^	15.33 ± 0.73^a^	3.70 ± 0.94^a^	395.88 ± 0.42^b^	86.67 ± 0.54^b^	278.57 ± 0.64^b^	45.71 ± 0.22^b^
Pea flour	144.25 ± 0.45^c^	7.49 ± 0.28^c^	1.67 ± 0.67^c^	408.46 ± 0.62^a^	245.44 ± 0.79^a^	235.65 ± 0.33^c^	33.77 ± 0.38^c^
Anchote flour	395.51 ± 0.57^a^	9.94 ± 0.83^b^	2.35 ± 0.45^b^	335.59 ± 0.83^c^	75.87 ± 0.99^c^	315.97 ± 0.85^a^	78.66 ± 0.76^a^

Note

Values are expressed as mean ± *SE*. Means not followed by the same superscript letters are significantly (*p* ≤ .05) different.

The mineral contents of the formulated diets are presented in Table 6. Mineral contents of the formulated diet ranged from 225.45 to 261.32, 11.48 to 12.61, 2.73 to 3.00, 357.92 to 391.13, 298.55 to 332.63, 252.00 to 278.01, and 44.26 to 51.56 for calcium, iron, zinc, phosphorous, potassium, sodium, and magnesium, respectively. Iron, zinc, phosphorus, magnesium, and calcium have been identified as the problem nutrients from 6 months of age and must be supplemented with the addition of complementary food (Bjelakovic, Nikolova, Gluud, Simonetti, & Gluud, 2007). The mineral content of the current study was almost within the range recommended by CODEX CAC/GL (1991) except iron and potassium contents (Table 6).

**Table 6:** Mineral contents (mg/100g, dwb) of six formulated complementary diets^a^

Formulated diets	Calcium	Iron	Zinc	Phosphorous	Potassium	Sodium	Magnesium
Diet 1	259.32	11.68	2.77	381.36	325.37	276.58	51.49
Diet 2	236.86	12.34	2.93	391.13	330.63	271.14	46.70
Diet 3	251.30	11.61	2.75	385.03	332.63	274.57	50.09
Diet 4	261.32	12.03	2.86	382.31	320.70	278.01	51.56
Diet 5	225.45	11.48	2.73	357.92	298.55	252.00	44.26
Diet 6	248.11	12.61	3.00	388.69	321.47	275.17	48.57
Codex standard^b^	250	16	3.2	356	516	296	32

^a^ Values are expressed as mean ± *SE*. Means not followed by the same superscript letters are significantly (*p* ≤ .05) different.

^b^ CODEX CAC/GL 08 (1991).

### 3.3 Mineral ratios of the formulated diets

The effectiveness of minerals in the diets is influenced by mineral–mineral interactions that may either enhance or reduce the absorption of certain micronutrients in the body (Soetan, Olaiya, & Oyewole, 2010). The awareness of such interactions, therefore, is useful when selecting vegetables that could help meet specific dietary criteria for improving micronutrient status. The mineral ratios are often more important than individual mineral levels themselves because they are useful in determining nutritional interrelationships and providing information regarding the many possible factors that may be represented by a disruption of their relationships such as disease states, physiological and developmental factors, and the effects of diets (Watts, 2010). The mineral ratios of the formulated diets are shown in Table 7.

**Table 7:** Mineral ratios of the six formulated complementary diets

Formulated diets	Na:K	Ca:P	Ca:K	Fe:Zn
Diet 1	0.107	0.680	0.797	4.217
Diet 2	0.114	0.606	0.716	4.212
Diet 3	0.109	0.653	0.755	4.222
Diet 4	0.106	0.684	0.815	4.206
Diet 5	0.112	0.630	0.755	4.205
Diet 6	0.111	0.638	0.772	4.203
Standard	<1	>0.5	<4	>2

The sodium–potassium (Na:K) ratios of the six formulated complementary diets ranged from 0.106 to 0.114. The recommended Na/K ratio should be less than one (Jacob et al., 2015). Ijarotimi, Adeoti, and Ariyo (2013) also reported that the Na/K ratio less than one is recommended for diets, particularly for hypertensive patients. Na/K ratio plays a very important role in the diet as it reduces high blood pressure and risk of stroke in the body (Jacob et al., 2015). According to Alinnor and Oze (2011), Na/K ratio is also of great importance for the prevention of high blood pressure if the Na/K ratio of the food value is less than one. The lower sodium and higher potassium intake help to reduce high blood pressure in hypertensive patients (Perez & Chang, 2014). Therefore, the observed Na/K molar ratio of the formulated complementary diets in this investigation revealed that consumption of these complementary diets would help to prevent hypertension and might lower blood pressure and may also be suitable for children who have the risk of high blood pressure.

The calcium–phosphorous (Ca:P) ratios of the six formulated complementary diets ranged from 0.606 to 0.684. The recommended Ca/P ratio should be >0.5 (Jacob et al., 2015). Furthermore, food is considered as good if Ca/P ratio is >1 and poor if this ratio is <0.5 (Alinnor & Oze, 2011). Chandran, Nivedhini, and Parimelazhagan (2013) also reported that the Ca/P ratio must be close to 1 for a good Ca and P intestinal utilization. A higher calcium–phosphorous (Ca/P) levels in foods are required for favorable calcium absorption in the intestine for bone formation (Adeyeye, Orisakeye, & Oyarekua, 2012). According to Adeoti et al. (2013), diets rich in protein and phosphorus may promote the loss of calcium in the urine. The Ca/P ratio in this study indicates that the formulated complementary diets would help calcium absorption in the body. The high Ca/P ratio observed in this study is of nutritional benefit, particularly for children and the aged who need higher intakes of calcium and phosphorus for bone formation and maintenance. It is well known that diets with a high value of Ca/P ratio are considered good, particularly for growing children who require a high intake of calcium and phosphorus for bone and teeth formation (Oluwole, Adeoti, & Ariyo, 2013).

The calcium–potassium (Ca/K) ratios of the six formulated complementary diets ranged from 0.716 to 0.815. The Ca/K ratio is called the thyroid ratio because calcium and potassium play a vital role in regulating thyroid activity (Olagbemide, Ojiezeh, & Adarabioyo, 2016). Low Ca/K ratio would indicate an elevation of thyroid expression (Watts, 2010). Since the Ca/K ratios of the formulated complementary diets are low as compared to the standard, the formulated diets are considered good for thyroid.

The iron–zinc (Fe/Zn) ratios of the six formulated complementary diets ranged from 4.203 to 4.217. Pérès, Bureau, Neuville, Arhan, and Bouglé (2001) reported that iron did not impair zinc absorption up to an iron: zinc ratio of 2:1; then a dose‐dependent effect was observed up to a ratio of 5:1; when the ratio was increased from 5:1 to 10:1, no further inhibition of zinc occurred. Based on this report, one can conclude that the iron present in the formulated complimentary diets did not impair zinc absorption.

### 3.4 Antinutritional contents of the raw ingredients and formulated diets

Table 8 shows the antinutritional contents of the ingredients. The pea flour was higher in phytate content and then followed by pea and anchote flours. Oxalate content of anchote flour was significantly (*p* < .05) higher than maize and pea flours. Tannin content of maize flour was significantly (*p* < .05) higher than pea and anchote flours. However, all the ingredients (maize, pea, and anchote) contain very low antinutritional contents. Antinutritional contents of the formulated diets are shown in Table 9. Phytate contents of the formulated diets ranged from 64.74 to 72.15 mg/100 g. The oxalate contents of the formulated diets ranged from 37.46 to 44.82 mg/100 g. Tannin contents of the formulated diets ranged from 10.41 to 11.77 mg/100 g. Antinutritional contents of the formulated diets are very low.

**Table 8:** Antinutritional contents (mg/100 g, dwb) of maize, pea, and anchote flours

Ingredients	Phytate	Oxalate	Tannin
Maize flour	67.37 ± 0.45^b^	32.29 ± 0.84^c^	14.65 ± 0.73^a^
Pea flour	96.00 ± 0.83^a^	42.73 ± 0.73^b^	9.38 ± 0.96^b^
Anchote flour	42.15 ± 0.71^c^	67.81 ± 0.84^a^	4.60 ± 0.63^c^

Note

Values are expressed as mean ± *SE*. Means not followed by the same superscript letters are significantly (*p* ≤ .05) different from each other.

**Table 9:** Antinutritional contents (mg/100 g, dwb) of the six formulated complementary diets

Formulated diets	Phytate	Oxalate	Tannin
Diet 1	67.37	44.82	10.41
Diet 2	72.15	40.06	11.77
Diet 3	69.98	44.15	10.58
Diet 4	67.32	44.10	10.72
Diet 5	64.74	37.46	10.74
Diet 6	69.70	40.74	11.28

Note

Values are expressed as mean ± *SE*. Means not followed by the same superscript letters are significantly (*p* ≤ .05) different.

### 3.5 Molar ratios and bioavailability of the formulated complementary diets

Bioavailability is the proportion of the total amount of mineral element that is potentially absorbable in a metabolically active form (Šimić et al., 2009). The calculated values of the molar ratios were also compared with the reported critical toxicity values for these ratios. Table 10 shows the calculated molar ratios of the formulated complementary diets.

**Table 10:** Calculated molar ratios of the six formulated complementary diets

Formulated diets	(Phytate:Ca)^a^	(Phytate:Fe)^b^	(Phytate:Zn)^c^	(Oxalate:Ca)^d^	(Phytate*Ca:Zn)^e^
Diet 1	0.016	0.049	2.41	0.079	0.157
Diet 2	0.018	0.049	2.44	0.077	0.144
Diet 3	0.017	0.051	2.52	0.080	0.158
Diet 4	0.016	0.047	2.33	0.077	0.151
Diet 5	0.017	0.048	2.35	0.076	0.132
Diet 6	0.017	0.047	2.30	0.075	0.143
Standard value	<0.24	>0.15	<10	<1	0.5

^a^ mg of phytate/molecular weight of phytate: mg of calcium/molecular weight of calcium.

^b^ mg of phytate/molecular weight of phytate: mg of iron/molecular weight of iron.

^c^ mg of phytate/molecular weight of phytate: mg of zink/molecular weight of zink.

^d^ mg of oxalate/molecular weight of oxalate: mg of calcium/molecular weight of calcium.

^e^ (mg of calcium/molecular weight of calcium) (mg of phytate/molecular weight of phytate)/(mg of zink/molecular weight of zink).

The molar ratios of phytate to calcium (Phy:Ca) ranged from 0.016 to 0.018. The critical molar ratio of [Phy]: [Ca] of <0.24 indicates good calcium bioavailability (Woldegiorgis, Abate, Haki, & Ziegler, 2015). The Phy:Ca molar ratios of the formulated diets in the present study were lower than the reported critical molar ratio, indicating the high absorption of calcium in all the formulated diets. Phytate to iron (Phy:Fe) ratios of the formulated diets varied from 0.047 to 0.051. The phytate:iron molar ratios >0.15 is indicative of poor iron bioavailability (Siegenberg et al., 1991). This result indicated that all the formulated diets contain the phytate:iron molar ratios of less than the critical value, this implies the high bioavailability and absorption of iron.

The molar ratios of phytate to zinc of the formulated diets varied from 2.30 to 2.52. The importance of foodstuffs as a source of dietary zinc depends on both the total zinc content and the level of other constituents in the diet that affect zinc bioavailability. The bioavailability of dietary zinc might be reduced by phytate (Bhandari & Kawabata, 2004). Hence, the Phy:Zn molar ratio is considered a better indicator of zinc bioavailability than total dietary phytate levels alone (Woldegiorgis et al., 2015). Foods with a molar ratio of Phy:Zn < 10 showed adequate availability of zinc, and there will be a problem encountered when the value is >15. Phy:Zn molar ratios > 15 is an indication of poor zinc bioavailability (Morris & Ellis, 1989). The values of the formulated complementary diets were lower than the critical molar ratios of Phy:Zn, which indicates the high bioavailability of zinc.

Oxalate to calcium (Ox:Ca) ratio of the formulated diets varied from 0.076 to 0.080. Oxalic acid and its salts can have deleterious effects on human nutrition and health, particularly by decreasing calcium absorption and aiding the formation of kidney stones (Bhandari & Kawabata, 2004). The importance of oxalate contents of an individual plant product in limiting total dietary calcium availability is of significance only when the ratio of Ox:Ca is greater than one (Frontela, Ros, & Martínez, 2009). From this result, it was observed that the Ox:Ca molar ratios of the formulated diets are lower than the reported critical value (1.0), which implies that oxalate cannot have any adverse effects on bioavailability of dietary calcium in these diets.

The molar ratios of phytate calcium to zinc ([Ca][Phy]/[Zn]) of the formulated diets varied from 0.132 to 0.158. The potent effect of calcium on zinc absorption in the presence of high phytate intakes has led to the suggestion that the [Phy][Ca]/[Zn] millimolar ratio may be a better index of zinc bioavailability than the [Phy]/[Zn] molar ratio alone (Frontela et al., 2009). High calcium levels in foods can promote the phytate‐induced decrease in zinc bioavailability when the [Ca][Phy]:[Zn] millimolar ratio exceeds 0.5 mol/kg (Adetuyi & Komolafe, 2011). In this study, the values of the formulated diets were lower than the critical molar ratios of [Ca][Phy]:[Zn], which indicates the high bioavailability of zinc in all the formulated diets.

## 4 CONCLUSION

In conclusion, good quality and acceptable complementary foods could be produced from maize, pea, and anchote flours. The proximate and mineral contents of the formulate diet 5 meet the Codex standards. The molar ratios of the formulated diets in this study were below standard reference and show high mineral bioavailability in all the formulated diets. The result of the study revealed that the formulated diets contain very low antinutritional factors and high mineral bioavailability. The paper's findings show that the complementary foods from maize, pea, and anchote flours particularly diet 5 may be suitable to alleviate protein energy malnutrition and it can be used as a substitute for the expensive commercial complementary food.

## CONFLICT OF INTEREST

Declared that no conflict of interest.

## ETHICAL APPROVAL

Human/animal testing is unnecessary in this study. Human subject is not involved in this study. Patients are also not involved in this study.
